# Reversal of English trend towards hospital death in dementia: a population-based study of place of death and associated individual and regional factors, 2001–2010

**DOI:** 10.1186/1471-2377-14-59

**Published:** 2014-03-26

**Authors:** Katherine E Sleeman, Yuen K Ho, Julia Verne, Wei Gao, Irene J Higginson

**Affiliations:** 1 Department of Palliative Care Policy & Rehabilitation, King’s College London, Cicely Saunders Institute, Bessemer Road, London SE5 9PJ, UK; 2 Public Health England Knowledge & Intelligence Team (South West), 149 Whiteladies Road, Bristol BS8 2RA, UK

**Keywords:** Dementia, Location of death, Terminal care, Hospitals, Nursing homes

## Abstract

**Background:**

England has one of the highest rates of hospital death in dementia in Europe. How this has changed over time is unknown. This study aimed to analyse temporal trends in place of death in dementia over a recent ten year period.

**Methods:**

Population-based study linking Office for National Statistics mortality data with regional variables, in England 2001–2010. Participants were adults aged over 60 with a death certificate mention of dementia. Multivariable Poisson regression was used to determine the proportion ratio (PR) for death in care home (1) and home/hospice (1) compared to hospital (0). Explanatory variables included individual factors (age, gender, marital status, underlying cause of death), and regional variables derived at area level (deprivation, care home bed provision, urbanisation).

**Results:**

388,899 deaths were included. Most people died in care homes (55.3%) or hospitals (39.6%). A pattern of increasing hospital deaths reversed in 2006, with a subsequent decrease in hospital deaths (−0.93% per year, 95% CI −1.08 to −0.79 p < 0.001), and an increase in care home deaths (0.60% per year, 95% CI 0.45 to 0.75 p < 0.001). Care home death was more likely with older age (PR 1.11, 1.10 to 1.13), and in areas with greater care home bed provision (PR 1.82, 1.79 to 1.85) and affluence (PR 1.29, 1.26 to 1.31). Few patients died at home (4.8%) or hospice (0.3%). Home/hospice death was more likely in affluent areas (PR 1.23, 1.18 to 1.29), for women (PR 1.61, 1.56 to 1.65), and for those with cancer as underlying cause of death (PR 1.84, 1.77 to 1.91), and less likely in the unmarried (PRs 0.51 to 0.66).

**Conclusions:**

Two in five people with dementia die in hospital. However, the trend towards increasing hospital deaths has reversed, and care home bed provision is key to sustain this. Home and hospice deaths are rare. Initiatives which aim to support the end of life preferences for people with dementia should be investigated.

## Background

Dementia care is a global health priority [1]. There are over 35 million people worldwide living with dementia, and this figure is predicted to double by 2030 [2]. In the United States, the total societal costs of dementia care are predicted to almost double by 2040 [3]. In Western Europe the prevalence of dementia is 6.9 per 100 people over 60, and total costs of dementia care account for an estimated 1.29% of GDP [2].

Dementia is under-recognised as a terminal illness [4]. Median survival following diagnosis is 2 to 4 years [5,6], and the trajectory of decline is characterised by progressive functional and cognitive deterioration, with acute illnesses such as infection frequently precipitating death [7]. Given the expanding population with the disease, providing good quality end of life care in dementia is an enormous challenge [8].

An understanding of where people die is essential to develop health policies aimed at improving end of life care. In addition, place of death can be an important indicator of the quality of end of life care [9]. In dementia, hospitalisation of people nearing the end of their lives can have a profound detrimental impact, with patients experiencing problems such as pressure sores, worsening of behavioural problems, and increased confusion [10]. Reducing the use of hospital-based care in advanced dementia also has potential economic benefits [11].

In the United States, there has been a recent reduction in hospital deaths, and an increase in home deaths, in both cancer and dementia [12]. In England, a similar pattern has been shown in cancer, likely in part due to implementation the National End of Life Care Programme in the mid-2000s which aimed to enable people to die in their preferred place (usually home) [13]. However, the National End of Life Care Strategy has been criticised for paying inadequate attention to the specific needs of people with dementia [14]. Data from 2003 showed that England had one of the highest rates of hospital death and lowest rates of home death in dementia in Europe [15]. How this has changed over time is not known. Therefore this study aimed to examine trends in place of death in dementia in England, and the individual and regional factors associated with place of death, over a recent 10 year period.

## Methods

### Design

Population-based cross-sectional study, 2001–2010 inclusive.

### Data sources

Mortality data for all deaths in England 2001–2010 were obtained from the Office for National Statistics (ONS). By law, all deaths in England must be registered within five days (unless a coroner’s inquest is necessary). Mortality data comprise information recorded on the death certificate including the date of death, age and gender of the patient, and the cause(s) of death, as well as information obtained by the Registrar’s Office at the time of death registration including marital status and address of residence. Since 1993 mortality statistics have recorded both the underlying cause of death, i.e. the disease that initiated the train of events leading to death, and contributory causes, defined as part of the causal sequence leading to death or contributing to death. Where a condition is listed as either the underlying cause of death, or a contributory cause, this is termed a mention.

These data were then linked at Lower Super Output Area (LSOA) level with regional variables including deprivation quintile, level of urbanisation, and care home bed provision. The LSOA is a geographic area designed for reporting small area statistics in England and Wales. There are 32,482 LSOAs in England, with an average population size of 1,500.

### Study population

All deaths from 2001–2010 where dementia was mentioned, either as the underlying or a contributory cause of death, were extracted using ICD-10 codes G30 (Alzheimers disease), F01 (vascular dementia), F03 (unspecified dementia) [16]. We chose to study all deaths with a mention of dementia (rather than only deaths from an underlying cause of dementia) since dementia is commonly a contributory cause of death, and the underlying cause of death is likely to be associated with place of death [17]. We focussed on cases aged over 60 (maturity onset dementia) [18]. Cases where the outcome variable (place of death) was unknown or classified as ‘other’ (e.g. in the street) were excluded.

### Variables

The outcome variable, place of death, was categorised as care home (includes nursing and residential homes), home, inpatient hospice (specialist palliative care inpatient units, either NHS or charitably funded), or hospital (includes NHS and private), based on routinely used ONS coding categories. Explanatory variables were individual demographic variables including age at death (analysed as an ordered categorical variable based on the data distribution, to aid comparison with previous studies: 60–79, 80–84, 85–89, 90–94, >95) [15,19], gender (men, women), marital status (married, single, divorced, widowed, unknown), year of death (2001–2005, 2006–2010), and underlying cause of death (grouped into dementia, chronic lower respiratory disease (ICD-10 J40-47), cancer (ICD-10 C00-97, D00-48), cardiovascular disease (ICD-10 I00-52, I70-99), cerebrovascular disease (ICD-10 I60-69), chronic neurological disease (ICD-10 G12 motor neuron disease, G20 Parkinsons disease, G35 multiple sclerosis) and ‘other’ (all remaining ICD-10 codes).

Regional variables (derived at LSOA level) were deprivation quintile (derived from the 2010 Index of Multiple Deprivation, an area-specific deprivation measure for LSOAs in England, 1 = most deprived, 5 = least deprived) [20], level of urbanisation (categorised as urban, semi-rural and rural based on the 2004 ONS Rural and Urban Area Classification) [21], and care home bed provision (information obtained from the Care Quality Commission in February 2012, analysed as an ordered categorical variable based on the data distribution: 0, 1–25, 26–50, >50).

### Statistical analysis

Percentages were used to describe the study population in terms of demographic and regional variables. The percentage of deaths in care home, hospital, home and inpatient hospice was standardised using the 2005–2010 mortality structure for more developed countries from the United Nations standard population [22], in order to allow comparison over time irrespective of age and gender changes. Trends were inspected visually, and linear regression with adjustment for age and gender was used to confirm trends in place of death over the time period.

Multivariable Poisson regression analyses was used to estimate proportion ratios (PR) for death in care home (1), or home/hospice (1), compared with hospital (0), for each of the variables studied. Home and inpatient hospice were grouped together since preliminary analyses showed similar trends in place of death for both, and numbers were small. Poisson regression was chosen in preference to logistic regression, since odds ratios do not provide an accurate measure of risk when applied to common outcomes [23]. A general estimating equation (GEE) method with exchangeable correlation matrix and robust 95% confidence intervals (CI) was included to account for clustering in the data at LSOA level. Explanatory variables (age, gender, marital status, underlying cause of death, IMD quintile, urbanisation, care home bed availability) were forced to stay in the model. For the home/hospice model, care home bed availability was not included as a variable since patients living in care homes would not be expected to die at home.

Analyses were performed using Stata version 10.

### Ethics and permission

This study was based on anonymised records, and no ethical approval was required according to national guidelines and those of King’s College London Research Ethics Committee. KS, YKH, WG and IJH were individually approved by ONS to analyse the data, through the ONS Data Access Agreement.

## Results

There were 397,513 deaths with a mention of dementia recorded in England between 2001 and 2010. These comprised 6.6% (95% CI 6.5 to 6.7) of all deaths in 2001, almost doubling to 12.0% (95% CI 11.9 to 12.1) of all deaths in 2010.

1,733 (0.4%) deaths at age less than 60, and 6,881 deaths with unknown place of death (1.7%), were excluded, leaving a total of 388,899 deaths included in subsequent analyses. Most deaths were women (66.9%); the mean age was 85.5 (SD 7.0) years. Most (60.2%) were widow(er)s, while just over a quarter (27.4%) were married. Most patients died in care home (55.3%) or hospital (39.6%). Very few deaths occurred at home (4.8%) or inpatient hospices (0.3%). Just under half of all deaths were certified with dementia as underlying cause of death (46.5%). These patients were more likely to be women, older, and die in care homes (Table 1).

**Table 1:** Individual and Regional characteristics of deaths with a mention of dementia (n = 388,899), an underlying cause of death of dementia (n = 180,905) and an underlying cause of death of non-dementia (n = 207,994) in people over 60 in England 2001-2010

**Variable**	**Value**	**All mentions dementia (N = 388,899)**		**Underlying cause of death: dementia (N = 180,905)**		**Underlying cause of death: non-dementia (N = 207,994)**	
			**%**		**%**		**%**
Gender	Men	128,684	33.1	52,194	28.9	76,490	36.8
	Women	260,215	66.9	128,771	71.1	131,504	63.2
Age mean (SD)		85.5 (7.0)		86.1 (7.1)		85.1 (6.8)	
Age group	60-79	70,987	18.3	29,991	16.6	40,996	19.7
	80-84	88,231	22.7	38,152	21.1	50,079	24.1
	85-89	113,768	29.3	52,079	28.8	61,689	29.7
	90-94	82,543	21.2	41,884	23.2	40,659	19.6
	>95	33,370	8.6	18,799	10.4	14,571	7.0
Marital status	Married	106,676	27.4	46,337	25.6	60,339	29.1
	Single	29,479	7.6	14,008	7.7	15,471	7.44
	Widowed	234,024	60.2	112,452	62.2	121,572	48.5
	Divorced	16,825	4.3	7,249	4.0	9,576	4.6
	Unknown	1,895	0.5	859	0.5	1,036	0.5
Underlying cause of death	Dementia	180,905	46.5	180,905	100.0	-	-
	Respiratory disease	8,662	2.2	-	-	8662	4.2
	Cancer	19,950	5.1	-	-	19,950	9.6
	Cardiovascular disease	43,953	11.3	-	-	43,953	21.1
	Cerebrovascular disease	68,888	17.7	-	-	68,888	33.1
	Chronic neurological	7,168	1.8	-	-	7,168	3.4
	Other	59,373	15.3	-	-	59,373	28.5
Year of death	2001-2005	166,553	42.8	80,395	44.4	86,158	41.4
	2006-2010	222,346	57.2	100,510	55.6	121,836	58.6
Deprivation quintile	1st (most deprived)	72,789	18.7	32,438	17.9	40,351	19.4
	2nd	78,982	20.3	36,124	20.0	42,858	20.6
	3rd	85,142	21.9	40,165	22.2	44,977	21.6
	4th	81,661	21.0	38,503	21.3	43,158	20.8
	5th (least deprived)	70,325	18.1	33,675	18.7	36,650	17.6
Urbanisation	Urban	309,179	79.5	142,618	78.8	166,561	80.1
	Semi-rural	39,967	10.3	19,199	10.6	20,768	10.0
	Rural	39,753	10.2	19,088	10.6	20,665	9.9
Care home beds/1,000	0	111,119	28.6	46,025	25.4	65,094	31.3
	1-25	75,359	19.4	34,748	19.2	40,611	19.5
	26-50	100,413	25.8	49,554	27.4	50,859	24.5
	>50	102,008	26.2	50,578	28.0	51,430	24.7
Place of death	Care home	215,183	55.3	115,120	63.6	100,063	48.1
	Hospital	153,916	39.6	57,086	31.6	96,830	46.6
	Home	18,670	4.8	8,489	4.7	10,181	4.9
	Inpatient hospice unit	1,130	0.3	210	0.1	920	0.4

SD: standard deviation.

People who died at home or in hospice were younger and more likely to be married than those who died in care home or hospital. More than half of deaths that occurred in hospice had cancer as underlying cause of death (Table 2).

**Table 2:** Individual and Regional characteristics of deaths with a mention of dementia occurring in care home (n = 215,183), hospital (n = 153,916), home (n = 18,670) and inpatient hospice unit (n = 1,130) in people over 60 in England 2001-2010

**Variable**	**Value**	**Place of death**									
		**Care home (N = 215,183)**		**Hospital (N = 153,916)**		**Home (N = 18,670)**		**Inpatient hospice unit (N = 1,130)**		**All (N = 388,899)**	
		**n**	**%**	**n**	**%**	**n**	**%**	**n**	**%**	**n**	**%**
Gender	Men	59,571	27.7	62,292	40.5	6,299	33.7	522	46.2	128,684	33.1
	Women	155,612	72.3	91,624	59.5	12,371	66.3	608	53.8	260,215	66.9
Age mean (SD)		86.1 (6.9)		84.9 (6.9)		84.3 (7.3)		81.8 (7.4)		85.5 (7.0)	
Age group	60-79	34,935	16.2	31,274	20.3	4,378	23.5	400	35.4	70,987	18.3
	80-84	46,180	21.5	37,210	24.2	4,538	24.3	303	26.8	88,231	22.7
	85-89	62,669	29.1	45,635	29.7	5,197	27.8	267	23.6	113,768	29.3
	90-94	49,353	22.9	29,749	19.3	3,311	17.7	130	11.5	82,543	21.2
	>95	22,046	10.3	10,048	6.5	1,246	6.7	30	2.7	33,370	8.6
Marital status	Married	47,570	22.1	49,925	32.4	8,629	46.2	552	48.9	106,676	27.4
	Single	17,441	8.1	11,251	7.3	730	3.9	57	5.0	29,479	7.6
	Widowed	139,925	65.0	84,988	55.2	8,665	46.4	446	39.5	234,024	60.2
	Divorced	9,209	4.3	6,954	4.5	592	3.2	70	6.2	16,825	4.3
	Unknown	1,038	0.5	798	0.5	54	0.3	5	0.4	1,895	0.5
Underlying cause of death	Dementia	115,120	53.5	57,086	37.1	8,489	45.5	210	18.6	180,905	46.5
	Respiratory disease	3,679	1.7	4,431	2.9	546	2.9	6	0.5	8,662	2.2
	Cancer	10,396	4.8	7,038	4.6	1,865	10.0	651	57.6	19,950	5.1
	Cardiovascular disease	20,774	9.7	20,539	13.3	2,569	13.8	71	6.3	43,953	11.3
	Cerebrovascular disease	38,902	18.1	26,791	17.4	3,092	16.6	103	9.1	68,888	17.7
	Chronic neurological	4,137	1.9	2,555	1.7	448	2.4	28	2.5	7,168	1.8
	Other	22,175	10.3	35,476	23.1	1,661	8.9	61	5.4	59,373	15.3
Year of death	2001-2005	95,395	44.3	63,900	41.5	6,933	37.1	325	28.8	166,553	42.8
	2006-2010	119,788	55.7	90,016	58.5	11,737	62.9	805	71.2	222,346	57.2
Deprivation quintile	1st (most deprived)	35,472	16.5	33,887	22.0	3,213	17.2	217	19.2	72,789	18.7
	2nd	42,343	19.7	32,984	21.4	3,435	18.4	220	19.5	78,982	20.3
	3rd	48,555	22.6	32,413	21.1	3,945	21.1	229	20.3	85,142	21.9
	4th	47,545	22.1	29,684	19.3	4,168	22.3	264	23.4	81,661	21.0
	5th (least deprived)	41,268	19.2	24,948	16.2	3,909	20.9	200	17.7	70,325	18.1
Urbanisation	Urban	165,574	77.0	128,469	83.5	14,167	75.9	969	85.8	309,179	79.5
	Semi-rural	23,757	11.0	14,079	9.2	2,051	11.0	80	7.1	39,967	10.3
	Rural	25,852	12.0	11,368	7.4	2,452	13.1	81	7.2	39,753	10.2
Care home beds/1,000	0	36,922	17.2	62,475	40.6	11,103	59.5	619	54.8	111,119	28.6
	1-25	39,432	18.3	31,676	20.6	4,003	21.4	248	22.0	75,359	19.4
	26-50	65,889	30.6	32,103	20.9	2,271	12.2	150	13.3	100,413	25.8
	>50	72,940	33.9	27,662	18.0	1,293	6.9	113	10.0	102,008	26.2

SD: Standard deviation.

**Figure 1:**
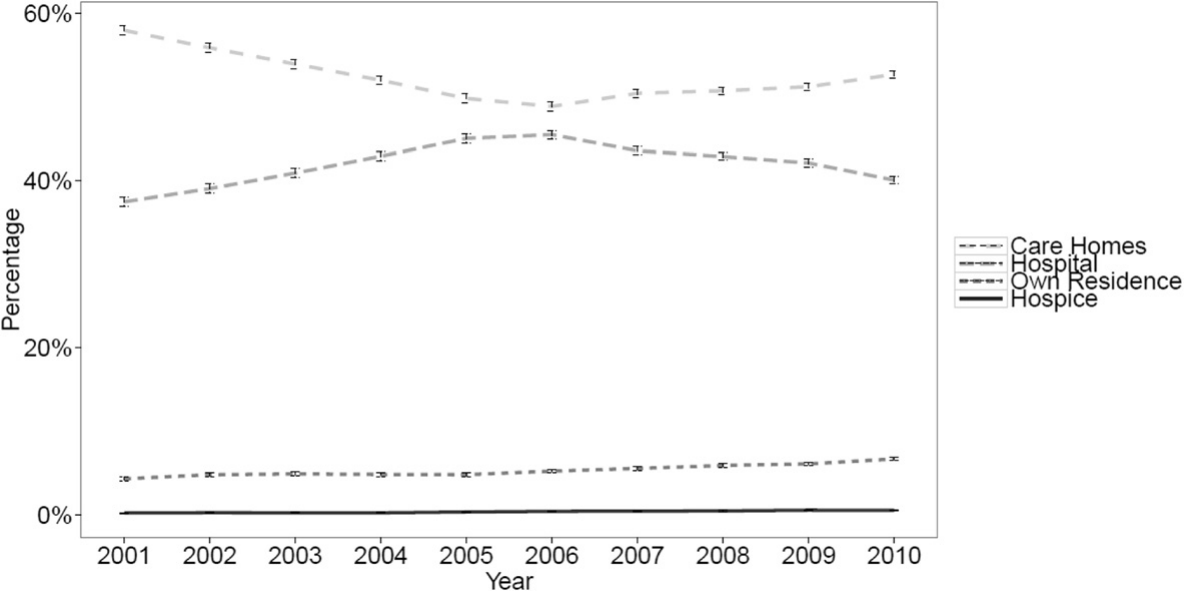
**Place of death for people with a death certificate mention of dementia in England 2001–2010.** Place of death given as percentage of deaths in care homes, hospital, own residence (home) and hospice with 95% CIs, age and gender-standardised against the UN mortality standard population [23].

As a proportion of all deaths, age and gender adjusted hospital deaths increased from 37.5% (95% CI 36.9 to 38.0) in 2001 to 45.4% (95% CI 44.9 to 46.0) in 2006 (1.91% per year, 95% CI 1.79 to 2.04 p < 0.001), and subsequently decreased to 40.1% (95% CI 39.6 to 40.5) in 2010 (−0.93% per year, 95% CI −1.08 to −0.79 p < 0.001). Reciprocally, care home deaths decreased from 57.9% (95% CI 57.4 to 58.5) in 2001 to 48.8% (95% CI 48.3 to 49.3) in 2006 (−2.06% per year, 95% CI −2.19 to −1.94 p < 0.001), and subsequently increased to 52.6% (95% CI 52.2 to 53.1) in 2010 (0.60% per year, 95% CI 0.45 to 0.75 p < 0.001). Home deaths increased from 4.4% (95% CI 4.1 to 4.6) in 2001 to 6.7% (95% CI 6.5 to 7.0) in 2010 (0.22% per year, 95% CI 0.20 to 0.25 p < 0.001), and inpatient hospice deaths increased very slightly from 0.3% (95% CI 0.2 to 0.3) in 2001 to 0.6% (95% CI 0.5 to 0.6) in 2010 (0.03% per year, 95% CI 0.03 to 0.04 p < 0.001) (Figure 1).

Living in an area with more care home beds per 1,000 population was the strongest factor associated with care home death (PR 1.82, 1.79 to 1.85). Likelihood of care home death was also higher for those living in affluent areas (PR 1.29, 1.26-1.31), those living in rural areas (PR 1.17, 1.15 to 1.19), those who were older (PR 1.11, 1.10 to 1.13) and women (PR 1.16, 1.16 to 1.17). Marital status did not strongly affect the likelihood of care home death. The likelihood of care home death was lower for four underlying causes of death: respiratory disease (PR 0.77, 0.75 to 0.79), cardiovascular disease (PR 0.80, 0.79-0.81), cerebrovascular disease (PR 0.92, 0.91-0.92), and ‘other’ underlying causes of death (PR 0.62, 0.62-0.63) (Table 3).

**Table 3:** Multivariable analysis: association of individual and regional characteristics with place of death (Care Home v Hospital and Home/Hospice v Hospital) in England 2001–2010

**Variable**	**Value**	**Care home v Hospital**			**Home/hospice v Hospital**		
		**PR**	**Lower 95% CI**	**Upper 95% CI**	**PR**	**Lower 95% CI**	**Upper 95% CI**
Age group	60-79	1.00	-	-	1.00	-	-
	80-84	1.00	0.99	1.01	0.92	0.88	0.95
	85-89	1.01	1.00	1.02	0.90	0.87	0.94
	90-94	1.05	1.04	1.06	0.94	0.91	0.98
	>95	1.11	1.10	1.13	1.05	0.99	1.11
Gender	Women v Men	1.16	1.16	1.17	1.61	1.56	1.65
Marital status	Married	1.00	-	-	1.00	-	-
	Single	1.01	1.00	1.02	0.51	0.48	0.55
	Divorced	0.97	0.95	0.98	0.65	0.60	0.69
	Widowed	1.01	1.00	1.02	0.66	0.64	0.68
	Unknown	1.03	0.99	1.07	0.59	0.47	0.74
Year of death	2001-2005	1.00	-	-	1.00	-	-
	2006-2010	0.96	0.96	0.97	1.24	1.20	1.27
IMD quintile	1st (most deprived)	1.00	-	-	1.00	-	-
	2nd	1.10	1.08	1.12	1.04	1.00	1.09
	3rd	1.17	1.14	1.19	1.09	1.04	1.14
	4th	1.21	1.19	1.24	1.16	1.11	1.22
	5th (most affluent)	1.29	1.26	1.31	1.23	1.18	1.29
Urbanisation	Urban	1.00	-	-	1.00	-	-
	Semi-rural	1.10	1.08	1.12	1.12	1.07	1.17
	Rural	1.17	1.15	1.19	1.52	1.46	1.59
Care home beds per 1000	0	1.00	-	-	-	-	-
	1-25	1.35	1.32	1.37	-	-	-
	26-50	1.68	1.65	1.70	-	-	-
	51-250	1.82	1.79	1.85	-	-	-
Underlying cause of death	Dementia	1.00	-	-	1.00	-	-
	Respiratory	0.77	0.75	0.79	0.87	0.80	0.94
	Cancer	1.00	0.98	1.01	1.84	1.77	1.91
	Cardiovascular	0.80	0.79	0.81	0.86	0.82	0.89
	Cerebrovascular	0.92	0.91	0.92	0.80	0.77	0.83
	Neurological	1.01	1.00	1.03	1.14	1.05	1.23
	Other	0.62	0.62	0.63	0.38	0.36	0.39

PR: proportion ratio.

PRs were estimated from Poisson regression models. The clustering effect within the LSOA geographical units was adjusted using the general estimating equation (GEE) method. A PR greater than 1 indicates higher probability of death in a care home or home/hospice.

The likelihood of home/hospice death was higher for women (PR 1.61, 1.56 to 1.65), and lower for those who were single, widowed or divorced compared to married (PRs 0.51-0.66). Areas with greater affluence and rural areas had higher likelihood of home/hospice death (PR 1.23, 1.18 to 1.29 and 1.52, 1.46 to 1.59 respectively). The likelihood of home/hospice death was higher where the underlying cause of death was cancer (PR 1.84, 1.77 to 1.91) and neurological disease (PR 1.14, 1.05-1.23). Age had little effect on likelihood of home/hospice death (Table 3).

## Discussion

This population-based study of place of death in dementia in England found that among people with a death certificate mention of dementia, hospital deaths remain amongst the highest in developed countries, with two in five people dying in hospital. However, the trend towards increasing hospital deaths in dementia reversed in 2006, with a subsequent fall in hospital deaths between 2006 and 2010, and a reciprocal increase in care home deaths. Care home bed provision and living in an area of least deprivation were the most important factors associated with care home death. Home and inpatient hospice deaths in dementia are rare, though both have increased slightly over time. An underlying cause of death of cancer, and being married were strongly associated with home/hospice death.

The reason for the shift from hospital to care home deaths in dementia is likely to be multifactorial. Policies such as the Community Care Act (2003), where financial incentives were introduced to prevent delayed hospital discharges, may have contributed. The importance of care home bed provision in promoting care home deaths is consistent with data from Europe and the United States [15,19], and there has been an increase in the number of nursing home beds (though not residential home beds) in England over this time period [24]. Overall, the proportion of hospital deaths in dementia is lower than the general population in England (58% 2005–2007), and care home deaths are higher in dementia than the general population (16% 2005–2007) [25].

39.6% of people with a death certificate mention of dementia died in hospital. In Europe, hospital deaths in dementia vary from 52.8% (Wales) to 22.8% (Belgium) [15]. In The Netherlands just 3.0% of dementia deaths occur in hospital, which may be due in part to the presence of specialised nursing home physicians, enabling 90.0% of patients with dementia to die in care homes [15]. In England and elsewhere the majority of nursing home care is provided by family physicians. Given the increasing need for high quality dementia care in care homes, alternative models of care (such as the Dutch model) should be considered.

Dying at home was rare (4.8% overall), though did increase slightly over the whole time period, suggesting that implementation of the UK National End of Life Care Programme in 2004 (a key aim of which was to enable people to die at home) had relatively little effect on home deaths in dementia. In contrast, home deaths in cancer increased from 22.4 to 25.8% over the same time period [13]. The UK End of Life Care Strategy (which was supported by the End of Life Care Programme) has been criticised for focussing on cancer, and containing inadequate reference to the growing number of people dying from dementia [14]. Our data support the need for initiatives which aim to meet end of life preferences for people with dementia.

A European study of place of death in dementia using data from 2003, found that home deaths varied from 3.3% (Wales) to 16.4% (Belgium) [15]. In the United States, home deaths in dementia are more common: home deaths increased from 19.9% in 2000 to 22.8% in 2009 in a cohort of Medicare beneficiaries with dementia [12], which coincided with an increase in use of the hospice benefit (community-based specialist palliative care) amongst this population from 19.5% to 48.3%. Enrolment in a hospice program has been shown to increase the likelihood of home death (and reduce hospital deaths) in patients with dementia in the United States [26], but whether community specialist palliative care support is an important factor enabling home deaths in dementia in England requires exploration. In England, community specialist palliative care input in dementia is currently uncommon [27], being most often associated with cancer. Accordingly, one of the strongest factors associated with home death in our study was an underlying cause of death of cancer. The likelihood of home death was also increased by marriage, which is consistent with studies in cancer and non-cancer [13,28], and indicates the importance of social support in facilitating home death.

In England, inpatient hospice units have been integral to the development of palliative care provision, and there are now 223 adult hospices in England providing inpatient and community care. In this study, very few people with a death certificate mention of dementia died in inpatient hospice units (0.3%, compared to 5% overall in England 2005–2007 [25]), and over half of hospice deaths in our study occurred in people who had an underlying cause of death of cancer. It is important to appreciate that this study can provide no information on the number of people with dementia who received support from specialist palliative care teams in the community (for example at home, or in care homes), though national data suggests fewer than 2% of people seen by community palliative care teams in England have dementia (compared with 80% with cancer diagnoses) [29]. The association between hospice death and cancer is consistent with previous studies [17].

In contrast to previous investigations of place of death in dementia [15,19], we studied all deaths with a mention of dementia, rather than only deaths from an underlying cause of dementia. More than half of deaths in the population were coded with an underlying cause other than dementia, and underlying cause of death was strongly associated with place of death. By using any mention of dementia, deaths from acute unforeseen events are included, which is important since these are patients in whom advance care planning can be most useful. It is important to be aware that a death certificate mention of dementia indicates that dementia was sufficiently advanced to contribute to death, whether or not it was the underlying cause. In cases where dementia had been diagnosed but was not thought to contribute to death, it would not be expected to appear on the death certificate.

This study used a whole population data set, allowing analysis of place of death, not limited by national generalisability. However, mortality data are limited by the number of variables available for analysis. For example, there was no information available on preference for place of death, trajectory of decline, symptom burden, or ethnicity, all of which influence place of death [28,30]. There was no information on health care transitions, aggressiveness of end of life care, or overall quality of care.

Studies using mortality statistics are susceptible to certification bias [31]. Dementia is known to be incompletely diagnosed in the UK, and even where it is diagnosed, it is underreported on death certificates. In one study dementia was not mentioned on the death certificate in 37% of people with known advanced dementia [32]. The influence of setting on death certification is unclear, though there is evidence that those who die in care homes are more likely to be certified with dementia as a cause of death, compared to other places of death [33]. Incentives to identify dementia as part of the primary care Quality and Outcomes Framework (QOF) may have increased certification of dementia over time, particularly amongst decedents in care homes where the majority of care is provided by general practitioners. For these reasons, care must be taken in interpretation that these data relate to people with a death certificate mention of dementia, rather than to the whole population.

## Conclusions

End of life care for the growing population with dementia is a public health priority. This study has provided high quality empirical data on where people with a death certificate mention of dementia die, and information regarding the factors associated with place of death, to inform health policies and planning. Important considerations for commissioners, policy makers and researchers are:

1. Home deaths remain extremely rare amongst people with a death certificate mention of dementia. Home palliative care services have been shown in meta-analysis to increase the odds of home death in cancer [34], but evidence in dementia is sparse [35], and high quality randomised controlled trials are needed.

2. Deaths amongst people with a death certificate mention of dementia in England have started to shift from hospitals to care homes. If this is to be maintained, care home bed provision must increase in line with the projected increase in dementia deaths.

3. Improved death certification is essential to fully understand place of death in dementia. The extent to which dementia certification practices are associated with place of death is unclear, and requires further exploration. Future studies using mortality statistics to explore place of death in dementia should take into consideration changes in certification practices over time.

## Abbreviations

LSOA: Lower super output area; GEE: Generalised estimating equation; PR: Proportion ratio; ONS: Office for National Statistics; ICD: International Classification of Diseases; IMS: Index of multiple deprivation; SD: Standard deviation; CI: Confidence interval.

## Competing interests

The authors declare that they have no competing interests. The views and opinions expressed therein are those of the authors and do not necessarily reflect those of the HS&DR programme, NIHR, NHS or the Department of Health.

## Authors’ contributions

WG, IJH, and JV obtained funding. KS, WG and IJH conceived the idea for this study and designed the analysis plan with input from all authors; KS contributed to the development of, and implemented this analysis plan with input from YKH; WG and YKH were responsible for liaison with the Office for National Statistics (ONS), data checking, recoding and merging of datasets. The manuscript was drafted by KS, with significant input from all authors. KS and IJH had full access to all the data in the study and take responsibility for the integrity of the data and the accuracy of the data analysis. All authors read and approved the final manuscript.

## Pre-publication history

The pre-publication history for this paper can be accessed here:

http://www.biomedcentral.com/1471-2377/14/59/prepub
